# An efficient method for disaster tweets classification using gradient-based optimized convolutional neural networks with BERT embeddings

**DOI:** 10.1016/j.mex.2024.102843

**Published:** 2024-07-03

**Authors:** Deepak Dharrao, Aadithyanarayanan MR, Rewaa Mital, Abhinav Vengali, Madhuri Pangavhane, Satpalsing Rajput, Anupkumar M. Bongale

**Affiliations:** a Department of Computer Science and Engineering, Symbiosis Institute of Technology, Pune Campus, Symbiosis International (Deemed University), Pune, India; b Department of Computer Engineering, Vishwakarma Institute of Technology, Pune, India; c Department of Artificial Intelligence and Machine learning, Symbiosis Institute of Technology, Pune Campus, Symbiosis International (Deemed University), Pune, India

**Keywords:** Natural language processing, CNN, Disaster tweets, Tweet classification, Deep Learning, BERT, *Disaster Tweet classification using CNN with BERT embeddings and RMS-Prop Optimization an Efficient Method for Disaster Tweets Classification using Gradient-Based Optimized Convolutional Neural Networks with BERT embeddings*

## Abstract

Event of the disastrous scenarios are actively discussed on microblogging platforms like Twitter which can lead to chaotic situations. In the era of machine learning and deep learning, these chaotic situations can be effectively controlled by developing efficient methods and models that can assist in classifying real and fake tweets. In this research article, an efficient method named BERT Embedding based CNN model with RMSProp Optimizer is proposed to effectively classify the tweets related disastrous scenario. Tweet classification is carried out via some of the popular the machine learning algorithms such as logistic regression and decision tree classifiers. Noting the low accuracy of machine learning models, Convolutional Neural Network (CNN) based deep learning model is selected as the primary classification method. CNNs performance is improved via optimization of the parameters with gradient based optimizers. To further elevate accuracy and to capture contextual semantics from the text data, BERT embeddings are included in the proposed model. The performance of proposed method - BERT Embedding based CNN model with RMSProp Optimizer achieved an F1 score of 0.80 and an Accuracy of 0.83. The methodology presented in this research article is comprised of the following key contributions:

• Identification of suitable text classification model that can effectively capture complex patterns when dealing with large vocabularies or nuanced language structures in disaster management scenarios.
• The method explores the gradient based optimization techniques such as Adam Optimizer, Stochastic Gradient Descent (SGD) Optimizer, AdaGrad, and RMSprop Optimizer to identify the most appropriate optimizer that meets the characteristics of the dataset and the CNN model architecture.
• “BERT Embedding based CNN model with RMSProp Optimizer” – a method to classify the disaster tweets and capture semantic representations by leveraging BERT embeddings with appropriate feature selection is presented and models are validated with appropriate comparative analysis.

## Graphical abstract

**Figure:**
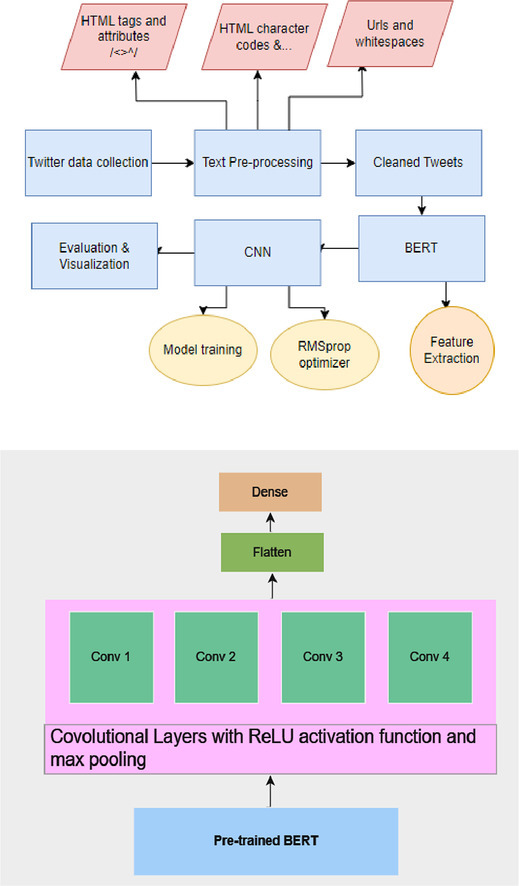

Specifications table

**Table:** 

Subject area:	*Computer Sciences*
More specific subject area	*Natural Language Processing*
Name of your method:	*Disaster Tweet classification using CNN with BERT embeddings and RMS-Prop Optimization an Efficient Method for Disaster Tweets Classification using Gradient-Based Optimized Convolutional Neural Networks with BERT embeddings*
Name and reference of original method:	*NA*
Resource availability:	*“Disaster Tweets”, Kaggle. Accessed on: Apr. 24, 2024. [Online]. Available:*https://www.kaggle.com/datasets/vstepanenko/disaster-tweets *https://github.com/deepakdharrao/Disaster-Tweets-Classification-using-CNN*

## Background

Disaster management is a comprehensive approach that encompasses preparedness, mitigation, response and recovery phases in order to minimize the adverse effects of natural or human-made disasters [1]. It requires careful planning, organization and coordination to save human lives, property and environment [2]. Disaster events and calamity news and information spreads expeditiously on social media platforms. The microblogging platform emerges as a valuable source of urgent narratives during emergencies. It captures the pulse of unfolding events, giving an avalanche of data to guide disaster response efforts. However, there can be so many tweets generated during crises that human analysts may find them overwhelming. At some times, such news could be falsified, incorrect or misleading and handling circulation of falsified news is crucial. This is where Natural Language Processing (NLP) becomes crucial by acting as a bridge whereby it employs different methods including sentiment analysis and information extraction to separate futile noise from important content [3,4]. The rapid and quick extraction of valuable and key information such as location and magnitude of an incident, needs expressed by affected populations, emergent discourses about disaster becomes crucial. Insights from the large amounts of textual data that inundates social media platforms is crucial during chaotic disastrous events [5]. NLP, which enables computers to understand and process human language is crucial in making sense out of what seems like random tweets during a crisis by identifying patterns, sentiments and trends contained within it that greatly improve disaster response strategies and decision-making processes [3,4].

We have developed Convolutional Neural Network (CNN) model using Bidirectional Encoder Representations from Transformers (BERT) embeddings and gradient-based optimization algorithms to solve the problem of differentiating between disaster-related tweets as either real or fake disasters [6,7]. The importance of contextual relevance such as in disastrous tweet text data [8,9] lies in its capacity to enhance the precision, accuracy, and interpretability of results [10,11]. BERT embeddings are a crucial part within our model. BERT is a cutting-edge language model that has been pre-trained on massive amounts of textual data. BERT embeddings are able to capture deep context information from text, making our model be aware of the fine points and complexities of language in a way that traditional word embeddings cannot. We integrate BERT embeddings into our CNN model, which combines the power of convolutional layers that captures local features well, and BERT's contextual understanding. This combination enhances tweet content analysis. Hence, there is some synergy between CNN and BERT embeddings that enables the model to differentiate between true disaster-related tweets versus non-true ones. The presented research methodology aims towards disaster tweet classification to enhance effective disaster response in the disaster-affected communities. The authors' investigations and salient contributions are summarized as follows:

(1) Implement the framework using BERT embedding for feature selection to improve the AI models.
(2) Explore various Optimization approaches.
(3) Evaluate and analyze the Logistic Regression, Decision Trees, and Convolutional Neural Networks (CNNs) for the purpose of classifying disaster-related tweets.

## Method details

**Fig. 1:**
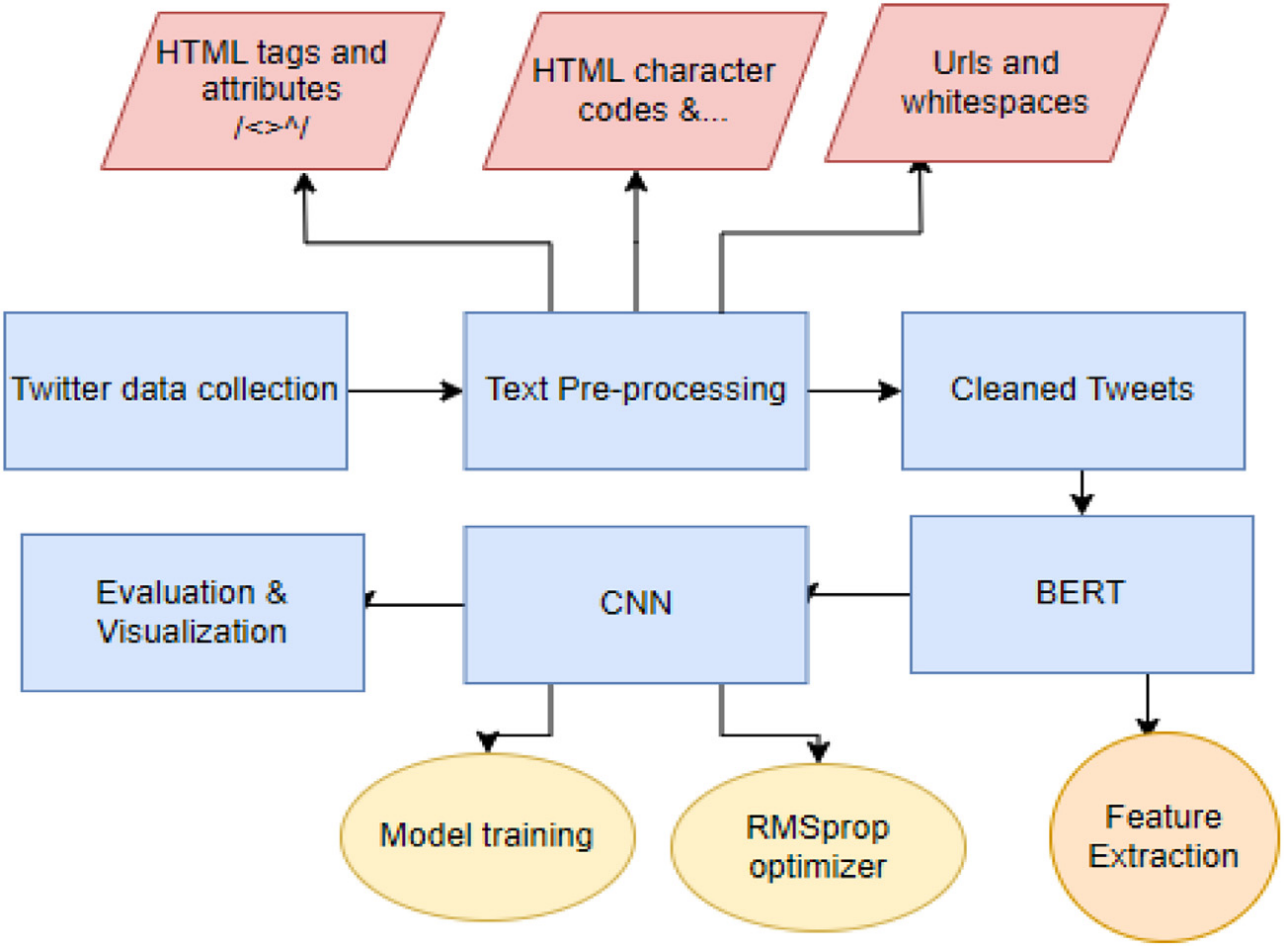
Block diagram illustrating the proposed methodology.

Our research study presents a methodology that uses a Convolutional Neural Network (CNN) architecture with BERT embeddings and an RMSprop optimizer to classify disaster tweets [12]. The CNN architecture's capacity to identify local patterns and hierarchies in textual data makes it a popular deep learning model for text categorization applications. This paper examines different machine learning and deep learning models for the task of disaster tweet classification and by analyzing the performance of these models we have selected CNN model for designing our proposed methodology with BERT embedding [13]. Through the presented research work, we aim at improving identification of tweets linked to serious emergencies from millions of social media updates. The proposed architecture and methodology is shown in Fig. 1. Rest of the subsection describes the dataset preparation and working of BERT embedding based CNN model with RMSProp optimizer for disaster tweet classification.

**Fig. 2:**
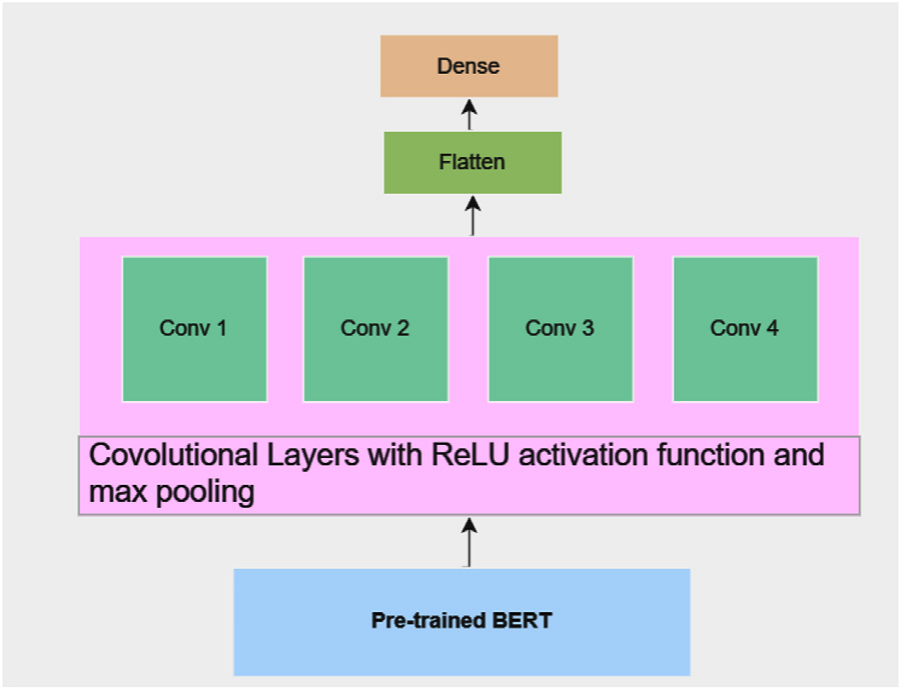
Proposed model layer architecture.

By incorporating BERT embeddings into CNN model, convolutional layers can significantly capture the local features and contextual understanding, which helps in analyzing the tweet content more holistically as shown Fig. 2. This synergy between CNN and BERT embeddings contributes to the model's ability to identify real disaster-related tweets from those that are not.

In the proposed architecture, the kernel size is set to 3 to capture local features in the text, such as trigrams (three-word combinations), which can be important for understanding the context and semantics of phrases. The model outcome predicts the probability distribution over two output classes, identifying disaster or non-disaster tweets. Categorical cross-entropy will penalize the model based on how far the predicted probability for "disaster" is from 1. The hyperparameters, including convolutional layer filter, kernel, dense units, dropout, and learning rate, are initially optimized using the random search function provided by *KerasTuner*. This involves defining ranges for each hyperparameter and training multiple models with different combinations to find the best-performing set. For instance, a learning rate of 0.001 was optimal for model training. Each hyperparameter plays a crucial role in shaping the model's performance. For example, a convolutional filter determines the number of filters in the first convolutional layer. At the same time, dropout regulates overfitting by randomly dropping neurons during training. The process aimed to maximize validation accuracy ensures the model's effectiveness in real-world scenarios. The various model-specific configuration of the CNN layers is mentioned in Table 1.

**Table 1:** Model specific configuration of the CNN layers.

Model Parameters	Values
Kernel size	3
Batch size	32
Number of layers	6
Number of filters	32
Loss function	Categorical cross-entropy
Dropout	0.5

### Dataset

The dataset used in this research article is fetched from the publicly accessible Kaggle platform [14]. The primary use case for this dataset is binary text classification. This dataset is a perfect fit for demonstrating disaster management scenario, hence the it is selected in the presented study. The dataset can be further utilized in various applications, including disaster monitoring, emergency response, and understanding public sentiment during disasters. The dataset is a CSV (Comma-Separated Values) file containing the following prominent feature:

*id:* A unique identifier for each tweet.

*text:* The content of the tweet.

*keyword:* A keyword from the tweet (often related to the disaster event, but not always). location: The location from which the tweet was sent (can be noisy or missing).

*target:* The binary target variable *if the* disaster tweet is real or fake text.

**Fig. 3:**
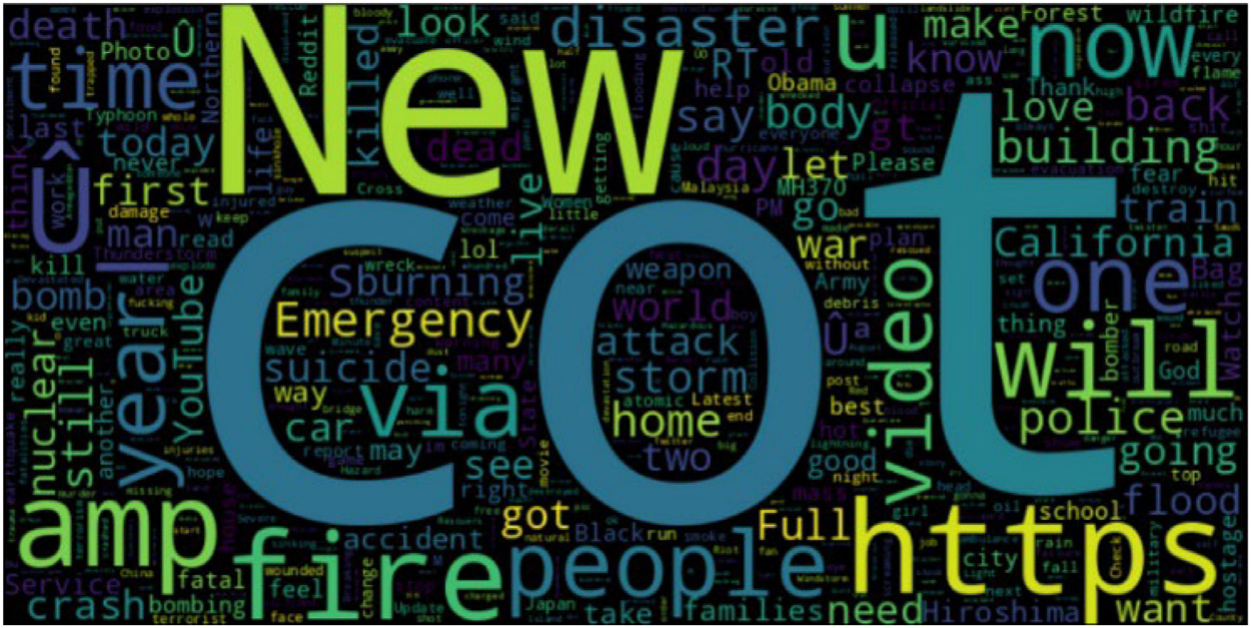
The most commonly used terms in the dataset of disaster tweets are displayed as a word cloud.

Before the model development, the text is preprocessed to eliminate the noisy data and imbalanced classes. Noisy data and unwanted data such as HTML tags, URLs, etc. are removed to make the cleaner text data. The word cloud visualization shown in Fig. 3 analyses the distribution of disaster-related words in the dataset and to summarize the most common terms. As can be seen from the word cloud in Fig. 3, tweets sent out during various catastrophes included phrases like fire, emergency, people, disaster, etc. that described the type and stage of the disaster.

### Machine learning and deep learning approaches

Primarily we experimented with classical machine learning algorithms as a baseline. The two most popular models used for classification were Logistic Regression and Decision Tree. While straightforward in design, these models offer valuable insights into the data, often rivaling the effectiveness of more complex alternatives [6].

*Logistic Regression:* This linear model analyzes the relationship between the features and the target labels, thereby predicting the likelihood of a tweet being related to a disaster [9,15]. While achieving moderate F1-score and accuracy scores, this model forms a vital benchmark for evaluating the efficacy of more intricate models. Logit function used in logistic regression as defined by the Eq. (1).

(1) $$f\left(z\right)=\frac{e^{z}}{1+e^{z}}=\frac{1}{1+e^{-z}},z\in R$$

*Decision Tree:* By recursively partitioning the feature space, Decision Trees identify distinctive patterns that guide tweet classification [16]. The analysis indicate improved comparable performance to the Logistic Regression model, underlining the complexity of the task due to the inherent noise and variability in disaster-related language. Entropy for one attribute is calculated by the Eq. (2).

(2) $$E\left(S\right)=\sum_{i=1}^{c}-p_{i}log_{2}p_{i}$$

Information gain is a decrease in entropy. More the information gain higher the level of node assigned to the decision. It is calculated by the Eq. (3).

(3) $$InformaionGain=Entropy\left(before\right)-\sum_{j=1}^{K}Entropy\left(j,after\right)$$

Gini index is a cost function to evaluate splits in dataset. It performs only binary splits. It is calculated by the Eq. (4).

(4) $$Gini=1-\sum_{i=1}^{c}{\left(p_{i}\right)}^{2}$$

Generally, machine learning models are good choice, but they fundamentally require manual feature engineering and may struggle to capture complex patterns when dealing with large vocabularies or nuanced language structures. This is where, deep learning models play pivotal roles. Recognizing the potential of deep learning models to capture intricate patterns, we delved into Convolutional Neural Networks (CNNs) - a class of models renowned for their prowess in image and sequence data analysis [17]. Leveraging various optimizers, we trained multiple CNN variations to discern the optimal architecture and hyperparameters for disaster tweet classification. The details are explained further below.

***CNN with Adam Optimizer:*** The role of the Adam Optimizer is to efficiently adjust the parameters of the neural network during the training phase. This is most suitable for tasks such as text classification. The parameters, such as moving averages of gradients and squared gradients, are adaptively fine-tuned to arrive at early convergence [18]. In the context of text classification of disaster tweets, exponentially decaying moving averages of gradients $\left(m_{t}\right)$ and squared gradients ($v_{t}$) are properly maintained. These are terms are generally tuned to compute bias-corrected estimates ($\hat{m_{t}}$ and $\hat{v_{t}}$). The convergence rate is adjusted via the learning rate (η) and then used to update the parameters ($\theta_{t+1}$) of the neural network. The associated mathematical equations are mentioned in the Eqs. (5), (6), (7), (8) and (9).

(5) $$m_{t}=\beta_{1}m_{t-1}+\left(1-\beta_{1}\right)g_{t}$$

(6) $$v_{t}=\beta_{2}v_{t-1}+\left(1-\beta_{2}\right)g_{t}^{2}$$

(7) $$\hat{m_{t}}=\frac{m_{t}}{1-\beta_{1}^{t}}$$

(8) $$\hat{v_{t}}=\frac{v_{t}}{1-\beta_{2}^{t}}$$

(9) $$\theta_{t+1}=\theta_{t}-\frac{\eta }{\sqrt{\hat{v_{t}}}+\epsilon }\hat{m_{t}}$$

The CNN architecture, coupled with the Adam optimizer, demonstrated superior performance in terms of F1-score and validation accuracy. This model captures intricate semantic relationships in disaster tweets, facilitating a more nuanced classification process.

***CNN with SGD Optimizer:*** Another well-known optimization technique namely, Stochastic Gradient Descent (SGD) Optimizer is used in the presented work [19]. The neural network parameters ($\theta_{t+1}$) are updated by computing the gradient of the loss function (f) during the training phase. In the context of disaster tweet classification, SGD facilitates iterative optimization towards minimizing the classification error by adjusting the parameters ($\theta_{t}$) for a single randomly chosen disaster tweet ($x_{i},y_{i}$). Similar to Adam Optimizer, here also learning rate (η) can be scaled appropriately. Large-scale text datasets such as disaster tweets are efficiently handled by considering the SGD optimizer. The mathematical representation is shown in the following Eq. (10).

(10) $$\theta_{t+1}=\theta_{t}-\eta \nabla f\left(\theta_{t};x_{i},y_{i}\right)$$

Despite exhibiting lower F1-score and validation accuracy compared to the Adam optimizer, the CNN model with Stochastic Gradient Descent (SGD) optimizer highlights the sensitivity of deep learning models to optimization techniques, necessitating careful selection based on the given data.

***CNN with AdaGrad Optimizer:*** AdaGrad is another optimizer know for fast convergence. Here the AdaGrad adjusts the neural network parameters ($\theta_{t+1,i})$, using a per-parameter learning rate, which is inversely proportional to the square root of the cumulative sum of squared gradients ($G_{t,ii}$) computed for each parameter during training. The nature of the disaster tweets where text data is sparsely distributed, the AdaGrad can provide excellent outcome. The adaptive learning mechanism assists in stabilizing the learning process and supports in reaching the ideal convergence of the neural network for accurate text classification [20]. Similar to the SGD variant, the AdaGrad optimizer yielded suboptimal results, emphasizing the significance of optimizer selection in achieving optimal convergence and generalization. The convergence and parameter updates in the neural network occur as per the Eqs. (11) and (12) shown below.

(11) $$G_{t,ii}=\sum_{\tau =1}^{t}g_{\tau ,i}^{2}$$

(12) $$\theta_{t+1,i}=\theta_{t,i}-\frac{\eta }{\sqrt{G_{t,ii}+\epsilon }}g_{t,i}$$

***CNN with RMSprop Optimizer:*** RMSprop Optimizer adjusts the neural network parameters using an exponentially decaying moving average of squared gradients ($G_{t}$). The neural network parameters ($\theta_{t+1}$) are adjusted during the training phase. The benefit of RMSProp lies in its effective adaptability characteristics to dataset such as disaster tweets text data. RMSProp considers classification tasks such as disaster tweets by providing stable updates to the parameters by scaling the learning rates (η) with the square root of the moving average and a small constant (ϵ). The CNN architecture with RMSprop optimizer showcased promising performance, outperforming other models in terms of both F1-score and validation accuracy. This underscores the potential of using adaptive learning rates for enhancing model performance. The mathematical aspects of RMSProp optimizer to update the neural network is shown below in Eq. (13) and (14).

(13) $$G_{t}=\rho G_{t-1}+\left(1-\rho \right)g_{t}^{2}$$

(14) $$\theta_{t+1}=\theta_{t}-\frac{\eta }{\sqrt{G_{t}+\epsilon }}g_{t}$$

**Fig. 4:**
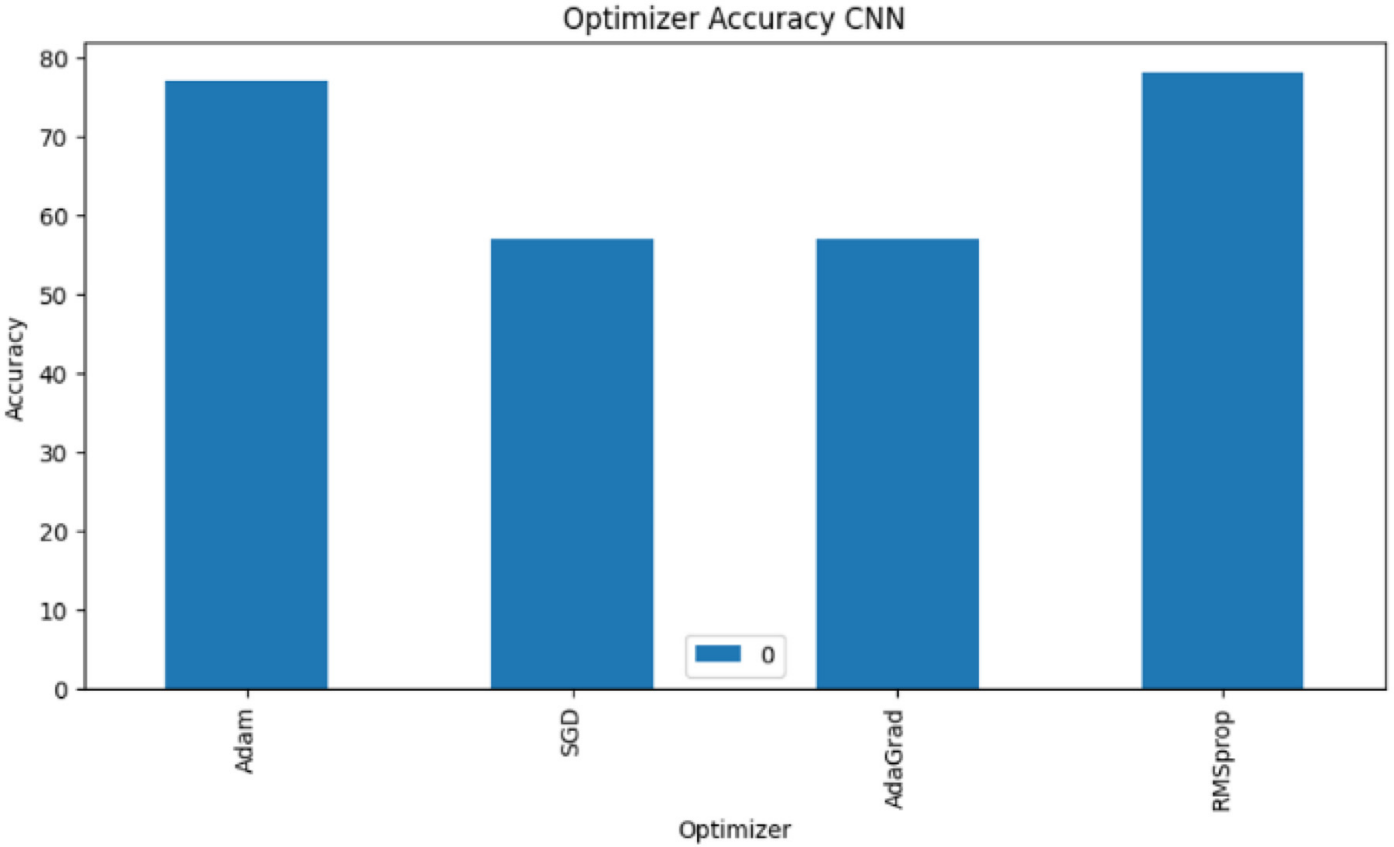
Accuracies of different optimizers with CNN model.

The adaptive learning rate of RMSprop optimizer in which it adjusts the learning rate for each parameter individually during training is one of its salient features [21]. Unlike AdaGrad, RMSprop does not suffer from decreasing learning rate in the presence of sparse data as it mitigates the issue by using exponential moving average of squared gradients. RMSprop has faster convergence in comparison to vanilla SGD and it also requires fewer hyperparameter tuning in comparison to AdaGrad and Adam. From the above described CNN variants with various optimizers, RMSprop optimizer is most convenient over other optimization techniques and the inference from Fig. 4 shows the superiority of the RMSProp optimizer in comparison to the rest.

### CNN with BERT

To further enhance the classification accuracy and to capture local features and patterns within text data, BERT embedding based CNN model with RMSProp optimizer is proposed. This model provides a deep understanding of contextual relationships in the tweet text data. We utilize BERT, a cutting-edge pre-trained big language model that collects phrase semantic meanings and contextual information, to integrate the input text. By refining the pre-trained BERT model on a sizable corpus of tweets on disasters, BERT embeddings are produced. Pretrained Language models have shown significant progress in recent years. The work presented in [22,23] shows comparisons of various pretrained modes for NLP tasks for text summarization, generation, and classification. Based on the study and review, BERT is identified as suitable pretrained model for disaster tweet classification.

To extract pertinent features from the input text, our implementation of the CNN comprises of many convolutional layers followed by max-pooling layers. Further, in order to classify these features, they are flattened and run through fully connected layers. This combination harnesses the strengths of both models, creating a hybrid that can effectively process and analyze text data. CNNs can efficiently extract features from the text, which can be further refined and contextualized by BERT. The proposed model gives promising results in terms of both F1-score and accuracy. The proposed model can automatically learn to discriminative characteristics from the input tweets.

In summary, this research extensively explored a spectrum of machine learning and deep learning models for disaster tweet classification. The incorporation of BERT embeddings provides a foundational representation of the data, while the CNN models, equipped with various optimizers, illustrate the impact of architecture and optimization choices on model performance. The insights derived from this study can guide the development of more sophisticated models for real-time disaster tweet classification, ultimately aiding emergency response efforts.

## Method validation

Here the performance of each model is discussed, drawing insights from the achieved accuracy and F1-score metrics. The comparative analysis of different models with optimizers is shown in Table 2.

**Table 2:** Comparison of different models.

Model	F1-score	Accuracy
Logistic Regression	0.50	0.53
Decision Tree	0.50	0.51
CNN with Adam Optimizer	0.75	0.76
CNN with SGD Optimizer	0.36	0.57
CNN with AdaGrad Optimizer	0.36	0.57
CNN with RMSprop Optimizer	0.76	0.78
BERT embedding based CNN model with RMSProp optimizer **(Proposed Methodology)**	**0.80**	**0.83**

The baseline machine learning models, Logistic Regression, and Decision Tree, provided a solid foundation for our investigation. The Logistic Regression model exhibited a macro-avg F1-score of 0.50 and an accuracy of 0.53, showcasing its capability to capture discernible patterns within the disaster tweet data. Similarly, the Decision Tree model, with a macro-avg F1-score of 0.50 and an accuracy of 0.51, demonstrated competitive results, emphasizing the feasibility of interpreting data through decision boundaries.

The exploration of Convolutional Neural Networks (CNNs) delved into the realm of deep learning and revealed a varying landscape of results based on different optimizers.

a. *CNN with Adam Optimizer:* The CNN model with the Adam optimizer excelled in terms of performance, achieving a macro-avg F1-score of 0.75 and a validation accuracy of 0.76. This outcome underscores the model's ability to capture intricate patterns within the textual data, resulting in improved classification accuracy for disaster tweets.
b. *CNN with SGD and AdaGrad Optimizers:* The CNN models equipped with SGD and AdaGrad optimizers both exhibited a lower macro-avg F1-score of 0.36 and validation accuracies of 0.57. This decrease in performance highlights the importance of selecting appropriate optimization techniques. The sensitivity of these models to the optimization process underlines the need for careful hyperparameter tuning to achieve optimal convergence and performance.
c. *CNN with RMSprop Optimizer:* The CNN architecture with the RMSprop optimizer performed relatively well in comparison to others in our study. It achieved a macro-avg F1-score of 0.76 and a validation accuracy of 0.78, signifying good adaptive learning rate and model's capacity to capture complex relationships within the disaster tweet data.
d. *CNN with BERT embeddings & RMSprop Optimizer:* The CNN architecture with BERT embeddings & RMSprop optimizer stood out as the top performer in our study. It achieved a macro-avg F1-score of 0.80 and a validation accuracy of 0.83, signifying the success of adaptive learning rate methods in enhancing the model's capacity to capture complex relationships within the disaster tweet data.

### Comparative analysis

**Fig. 5:**
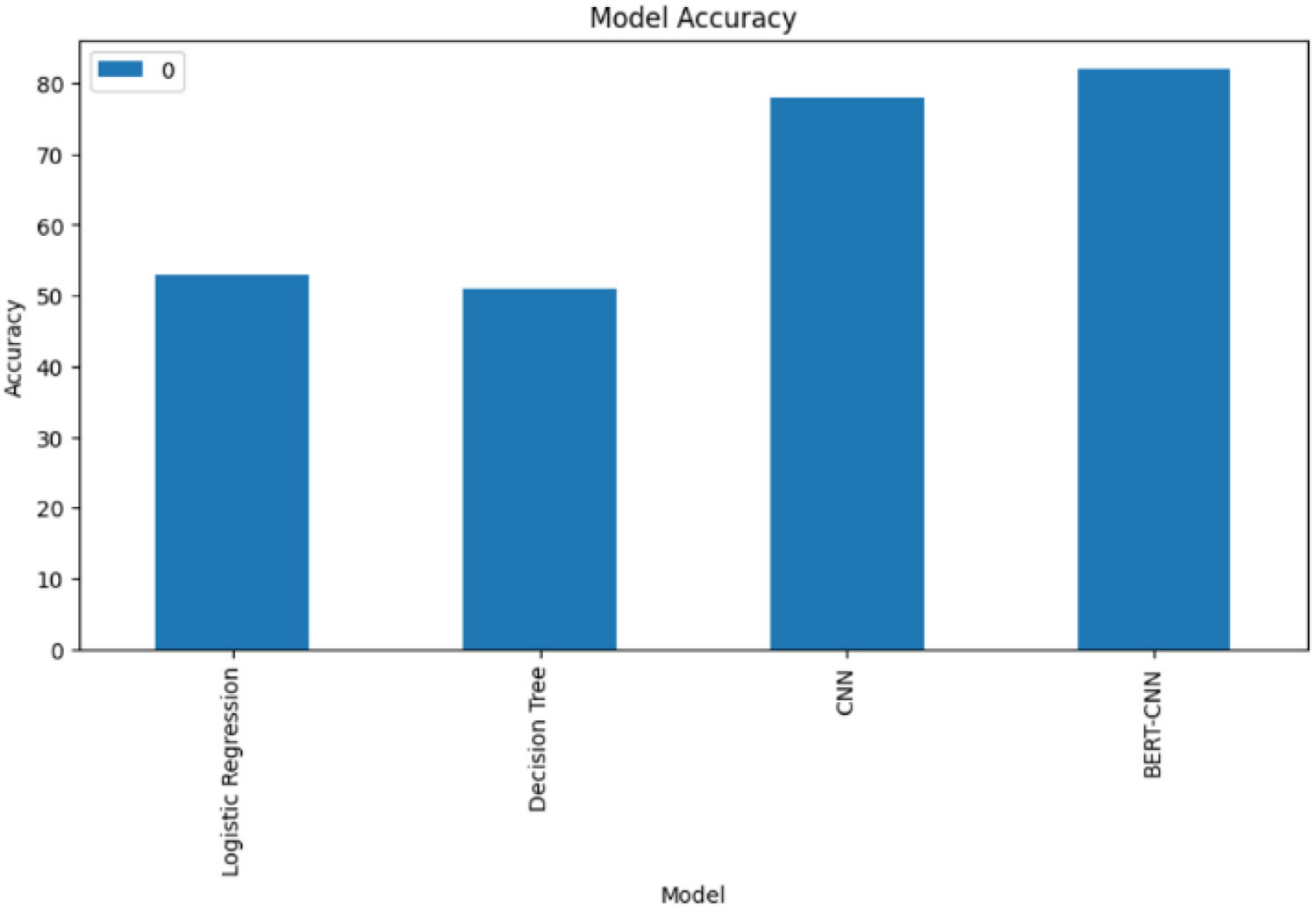
Accuracies of ML and DL models in classifying disaster.

The deep learning models, particularly the BERT-CNN model utilizing the RMSprop optimizer, outperformed the traditional machine learning approaches in both F1-score and accuracy metrics. This outcome emphasizes the power of deep learning in uncovering intricate patterns and relationships within unstructured textual data, which is particularly relevant for disaster tweet classification where context and nuance are crucial. The inference from Fig. 5. depicts the superiority of the CNN model in comparison to the other ML approaches and hence we use the optimizers to increase its performance.

**Fig. 6:**
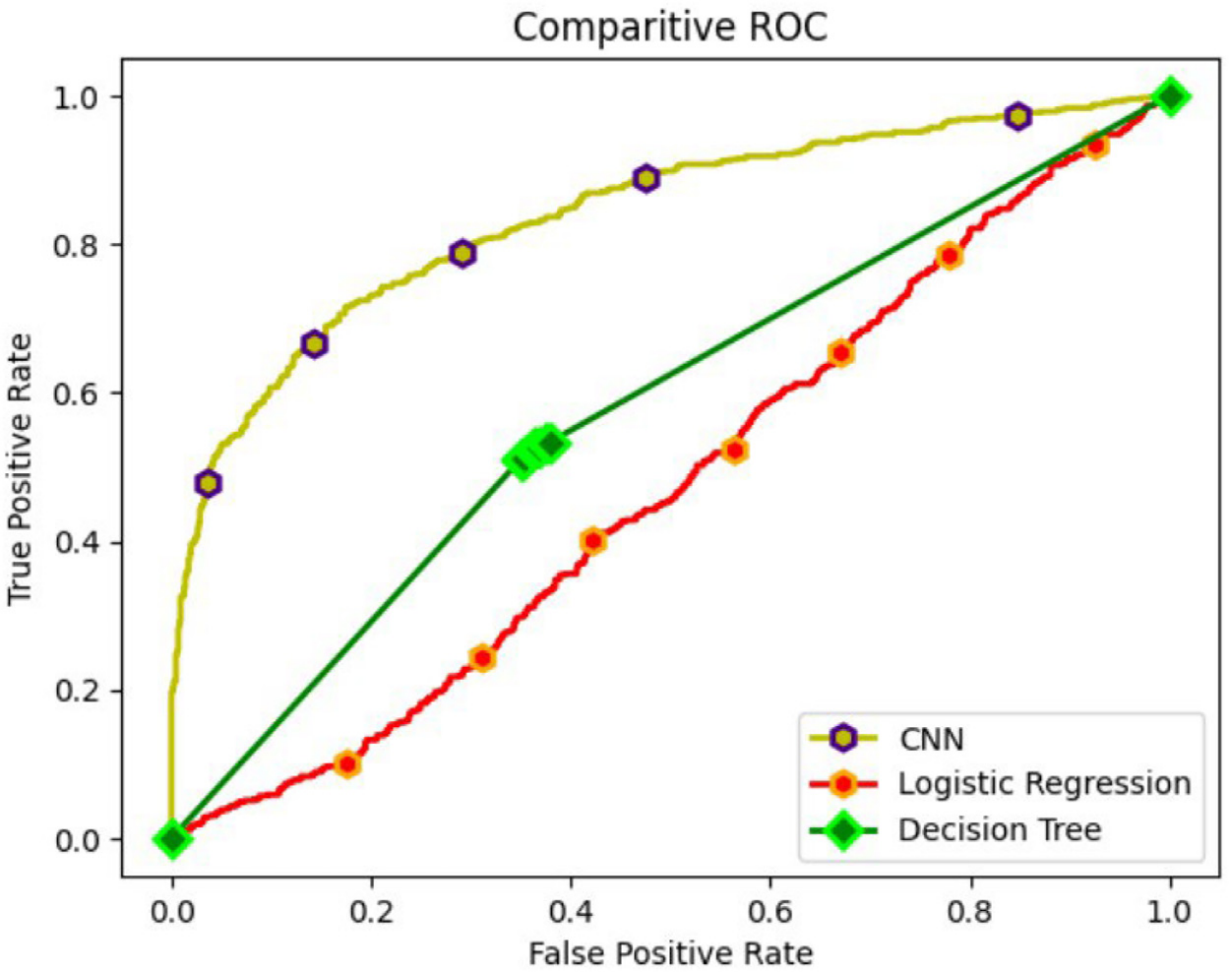
ROC curve analysis of different models.

Fig. 6 showing comparative analysis using ROC curves of the models revealed a stark contrast among the models. The CNN curve soared confidently to the upper-left corner, signifying its outstanding sensitivity and minimal false positives. In contrast, the Decision Trees curve, while second best, trailed noticeably behind, possibly due to challenges in handling complex textual data. Logistic Regression, the least effective, featured a curve that closely paralleled the diagonal line, indicating its limited discriminatory power in disaster tweet classification.

**Fig. 7:**
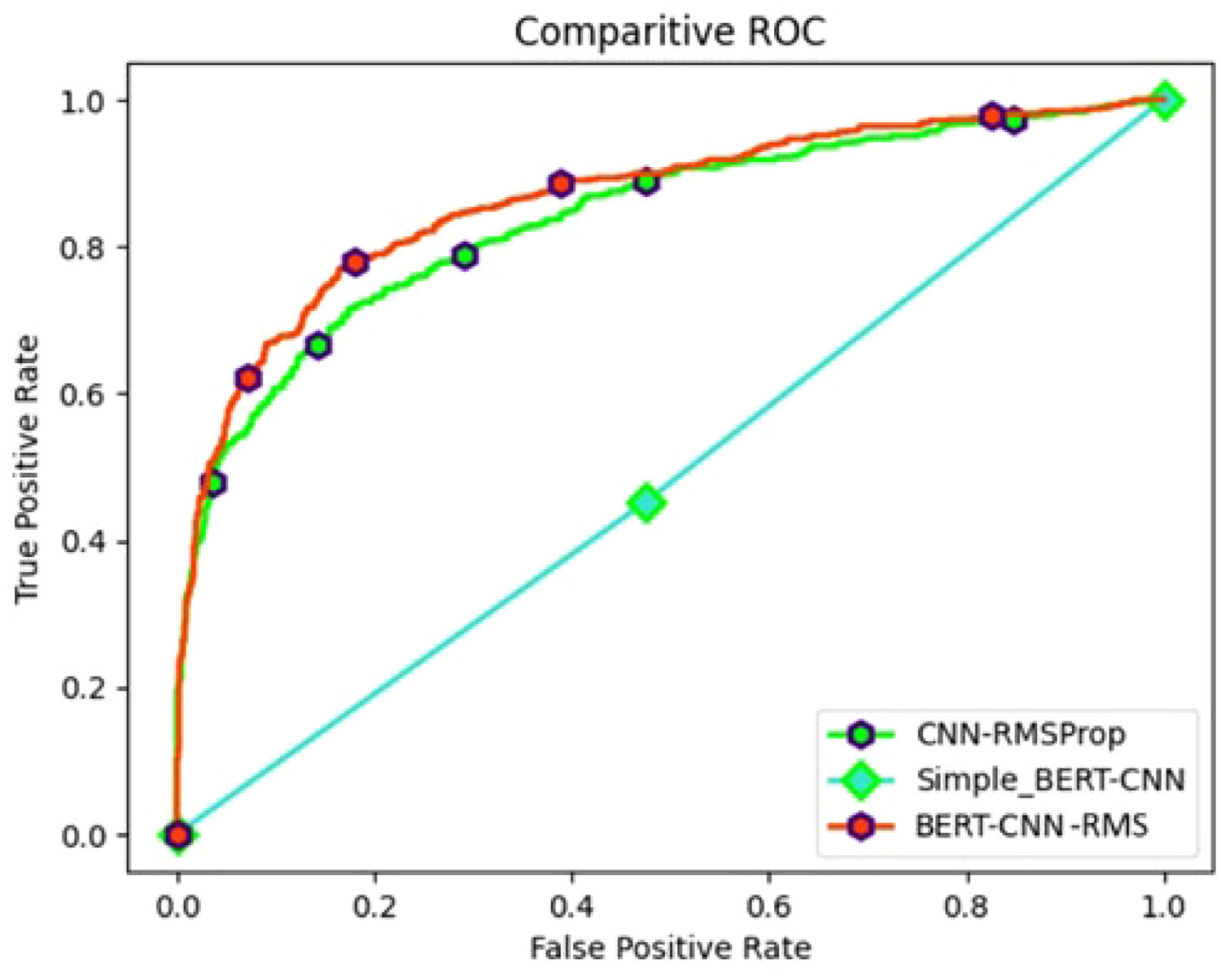
ROC curve analysis of CNN models.

Fig. 7 shows ROC curve analysis between BERT-CNN with RMSprop, CNN with RMSprop and simple BERT-CNN. The superiority of BERT-CNN with RMSprop is evident as it gives the best curve hugging the upper-left corner, indicating outstanding sensitivity and minimal false positives. This model's superior performance is further underlined by its ability to effectively distinguish between disaster-related and non-disaster-related tweets.

In comparison, the CNN with RMSprop demonstrates very good performance as it is very similar to the BERT-CNN with RMSprop curve, only slightly falling short of it. However, the BERT-CNN with RMSprop outperforms it in terms of sensitivity and discriminatory power, highlighting the advantages of integrating BERT embeddings and optimizing with the RMSprop algorithm. The simple BERT-CNN gives a really poor result which is simply a straight slope.

The computational efficiency of the model, specifically the BERT-CNN-RMSProp architecture, is notable, with a training time of merely 18 s on a T4 GPU for 10 epochs, averaging 7–11 milliseconds per step. Similarly, the inference time stands impressively at 5 milliseconds per step. To optimize these performance metrics further, employing well-defined models and leveraging GPUs are paramount. Well-defined models ensure streamlined processing, while GPUs significantly reduce training time by parallelizing computations. This optimization strategy not only enhances efficiency but also facilitates rapid model iteration and deployment, crucial for real-time applications.

To summarize, Fig. 4 displays the performance of the CNN model using the Adam, SGD, AdaGrad, and RMSprop optimization methods. The accuracy of the four models—LR, DT, CNN, and BERT-CNN as measured by the disaster tweets dataset is shown in Fig. 5. When given the data, the deep learning models perform better in terms of accuracy. As observed from the ROC curve in Fig. 6 and Fig. 7, it is evident that the BERT embedding based CNN model with RMSProp optimizer is superior at classifying disaster tweets.

## Conclusion

In conclusion, the presented research comprehensively examines the effectiveness of diverse machine learning and deep learning models for disaster tweet classification. The CNN models, particularly those employing the BERT embeddings and RMSprop optimizer, displayed remarkable performance improvements over traditional machine learning approaches. These findings emphasize the potential of deep learning techniques in accurately identifying disaster-related tweets, which in turn can contribute to more effective and efficient emergency response efforts. The proposed model - “BERT embedding based CNN model with RMSProp optimizer”- has an accuracy of 0.83, which is comparatively better than other machine learning and deep learning models. Though the proposed model can provide prominent accuracy, there are a couple of avenues for future extension of the work. Hybrid models combining BERT with other NLP architectures with real-time data processing systems can be considered for further enhancement of model accuracy.

## Limitations

Not applicable.

## Ethics statements

There were no animal experiments performed or animal subject used in the research work presented in this paper. Dataset obtained from twitter and twitch application are obtained from KAGGLE, an open-source platform. Hence, the KAGGLE's data redistribution policies were complied with.

## CRediT authorship contribution statement

**Deepak Dharrao:** Writing – original draft, Writing – review & editing, Investigation, Supervision. **Aadithyanarayanan MR:** Methodology, Writing – original draft, Conceptualization. **Rewaa Mital:** Writing – original draft, Conceptualization. **Abhinav Vengali:** Software, Writing – original draft, Data curation, Validation. **Madhuri Pangavhane:** Writing – original draft, Writing – review & editing, Investigation, Validation. **Satpalsing Rajput:** Writing – original draft, Writing – review & editing, Validation. **Anupkumar M. Bongale:** Writing – original draft, Formal analysis, Project administration, Resources.

## Declaration of competing interest

The authors declare that they have no known competing financial interests or personal relationships that could have appeared to influence the work reported in this paper.

## Data Availability

Data will be made available on request.
